# Analysis of muscle synergy and gait kinematics during regain of gait function through rehabilitation in a monoplegic patient

**DOI:** 10.3389/fnhum.2023.1287675

**Published:** 2024-01-09

**Authors:** Akira Ebihara, Mitsuki Hirota, Yasuhiro Kumakura, Masanori Nagaoka

**Affiliations:** ^1^Department of Rehabilitation, Tsubasa-no-ie Hospital, Oyama, Tochigi, Japan; ^2^Department of Rehabilitation Medicine, Dokkyo Medical University, Shimotsugagun, Tochigi, Japan; ^3^Division of Rehabilitation Services, Tsubasa-no-ie Hospital, Oyama, Tochigi, Japan; ^4^Department of Neurology and Rehabilitation, Graduate School of Medicine, Juntendo University, Tokyo, Japan

**Keywords:** stroke, monoplegia, muscle synergy, non-negative matrix factorization, gait kinematics, recovery process, number of strides

## Abstract

**Purpose:**

We conducted muscle synergy and gait analyses in a monoplegic patient whose gait function improved through training, to explore the possibility of using these parameters as indicators of training.

**Case presentation:**

A 49-year-old male had monoplegia of the right lower limb caused by infarction of the left paracentral lobule. After 2 months of training, he was able to walk and returned to work.

**Methods:**

Consecutive analyses were done after admission. Muscle synergy analysis: during walking, surface electromyograms of gluteus maximus, quadriceps femoris, adductor femoris, hamstrings, tibialis anterior, medial/lateral gastrocnemius, and soleus on both sides were recorded and processed for non-negative matrix factorization (NNMF) analysis. Gait analysis: markers were placed at foot, and walking movements were video recorded as changes in position of the markers.

**Results:**

Compared with three muscle synergies detected on the non-paretic side, two muscle synergies were extracted on the paretic side at admission, and the number increased to three and then four with progress in rehabilitation training. Changes in weighting and activity of the muscle synergies were greater on the non-paretic side than on the paretic side. With training, the knee joint flexor and the ankle dorsiflexor activities on the paretic side and the gluteus maximus activity on the non-paretic side increased during swing phase as shown by weight changes of muscle synergies, and gait analysis showed increased knee joint flexion and ankle joint dorsiflexion during swing phase in the paretic limb. On the non-paretic side, however, variability of muscle activity was observed, and three or four muscle synergies were extracted depending on the number of strides analyzed.

**Conclusion:**

The number of muscle synergies is considered to contribute to motor control. Rehabilitation training improves gait by increasing the number of muscle synergies on the paretic side and changing the weights of the muscles constituting the muscle synergies. From the changes on the non-paretic side, we propose the existence of compensatory mechanisms also on the non-paretic side. In muscle synergy analysis, in addition to the filters, the number of strides used in each analysis set has to be examined. This report highlights the issues of NNMF as analytical methods in gait training for stroke patients.

## 1 Introduction

In providing rehabilitation for hemiplegic patients with the goal to regain gait, a common clinical question is: what should be used as the indicator of training? One approach is to focus on the muscle strength of the paretic muscles in the early stage of disease when flaccid paralysis is prominent; to promote the extensor muscle activity of the trunk for maintaining a sitting or standing posture, or to promote tone enhancement of the extensor muscles in the case of lower extremities. Conversely, during the stage of intensified spasticity, techniques to control spasticity are used. Another approach that has been employed is to realize actual walking using orthoses in patients with muscle tone abnormalities. Some studies used methods including monitoring leg muscle strength and measuring center of gravity using a stabilometer (Nakamura et al., 1992; Suzuki et al., 1999). Recent studies have used robotic devices to change parameters during walking, with the goal to improve gait (Tan et al., 2020; Ishibashi et al., 2021). Considering the treatment of gait disturbance after stroke, the relation of muscles and their kinematics should be explored. Knutsson and Richards (1979) analyzed the gait disturbance in stroke patients using electromyography (EMG) and he mentioned the importance of the knowledge of both the actual gait movement and EMG activities of leg muscles for rehabilitation (Knutsson, 1994), but did not directly propose functional training methods. Moreover, there are few methods using conventional EMG recording which provide feedback of the results during training in the clinical setting. The availability of visible indicators that can be analyzed within a short time and are fed back during training will allow implementation of more effective rehabilitation.

In human movements, the brain does not control the activities of large numbers of muscles independently, but controls groups of muscles (synergies) to execute complex movements (Bernstein, 1967). Muscle synergies in hemiplegic patients have been reported in recent years (Clark et al., 2010; Kautz et al., 2011; Allen et al., 2013; Hashiguchi et al., 2015, 2016). The number of muscle synergies has been reported to decrease in hemiplegia, and the number increases with recovery of gait function (Routson et al., 2013). However, there are some debates on the methodology of muscle synergy analysis, such as the filters used to process surface EMG, number of strides to be analyzed, and the type of dataset (averaged or concatenated).

In this study, we investigated the neural mechanisms involved in gait by performing muscle synergy analysis in a patient with focal infarction in the cerebral cortex of the frontal lobe (paracentral lobule) and analyzing the recovery process of lower limb paresis.

## 2 Case presentation

A 49-year-old male had right lower limb monoplegia caused by focal infarction of the left paracentral lobule. The patient was unable to voluntarily contract the distal muscles of the right lower limb, although he was capable of unsteady gait. While he apparently walked as automatic movements, he was not able to contract individual lower limb muscles. This case was previously reported as a case of automatic-voluntary dissociation (Nagaoka et al., 2022). No sensory disturbance was detected. Although lower limb tendon reflexes were reduced at admission, the reflexes increased mildly during hospitalization. After approximately 2 months of rehabilitation training, although he remained incapable of voluntarily contracting individual lower limb distal muscles (triceps surae, tibialis anterior), his stride length improved from 986.3 to 1,456.6 mm and cadence from 92.4 to 105.6 steps/min, and he was able to walk outdoors. Therefore, he was discharged from the hospital and subsequently returned to his former job.

## 3 Materials and methods

### 3.1 Walking task

Measurements were performed for a total of four times during the patient’s course of hospitalization: at admission, twice during hospitalization, and at discharge (Table 1). The walking tasks were as follows. For the first measurement (10 days after admission), the patient walked barefoot on the floor at a self-selected speed. From the second measurement onward, the patient walked on a treadmill at a speed of 2 or 3 km/h (33 m/min or 50 m/min) both with and without using a handrail. We did not use a safety harness or body weight unloading, but to prevent falling, one physiotherapist supervised the patient closely during walking, and the patient was allowed to hold on to the handrail in case of emergency. On the treadmill, the patient wore a sock on his right foot to lessen the friction between the foot and treadmill belt. In addition, measurement was also performed with the patient walking while wearing a plastic ankle-foot orthosis (AFO) on the paretic lower limb. Table 1 details the walking conditions and maximum numbers of strides obtained in the four analyses.

**TABLE 1:**
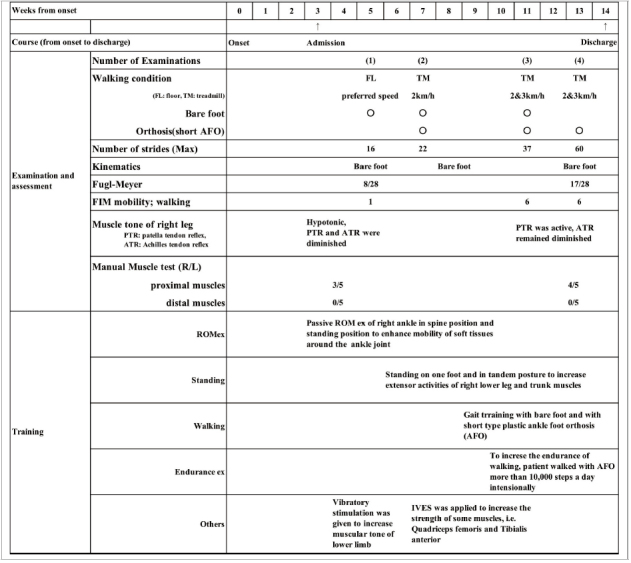
Clinical course from onset of stroke to discharge and details of assessments and training.

During hospitalization, we conducted EMG and video recordings four times, and kinematic analyses three times. The conditions of walking are shown. Some examinations were conducted under bare foot and AFO conditions. For other trainings, we applied vibrator to his right leg aiming to enhance the spinal stretch reflex and increase muscle tone. At the second examination, we noticed that the patient tended to have shorter strides during the latter half of the gait cycle, because of the tendency of knee flexion. We sometimes used IVES to increase muscle power of the muscles. The flexion pattern was improved in the third examination. IVES, integrated volitional control electrical stimulation.

### 3.2 Simultaneous EMG and video recordings

While the patient was walking, a Bagnoli Desktop System (Delsys Inc., Boston, MA, USA) was used to record surface EMG on right and left gluteus maximus (Glut), quadriceps femoris (Quad), adductor femoris (Add), hamstrings (Hamst), tibialis anterior (TA), lateral and medial gastrocnemius (GLat and Gmed), and soleus (Sol). A total of 16 electrodes were placed on the muscles with double-sided adhesive skin interfaces. Using the Teraview^®^ ver. 1.3.49 software (Medical Try System, Tokyo, Japan) (Sato et al., 2007) the EMG were integrated with the video data recorded simultaneously during walking with a digital video camera (Panasonic NV-GS300, 30 fps). From the video images, heel strike was visually determined for each gait cycle (comprising heel strike, stance, swing, next heel strike).

### 3.3 Methodology of muscle synergy analysis

**FIGURE 1:**
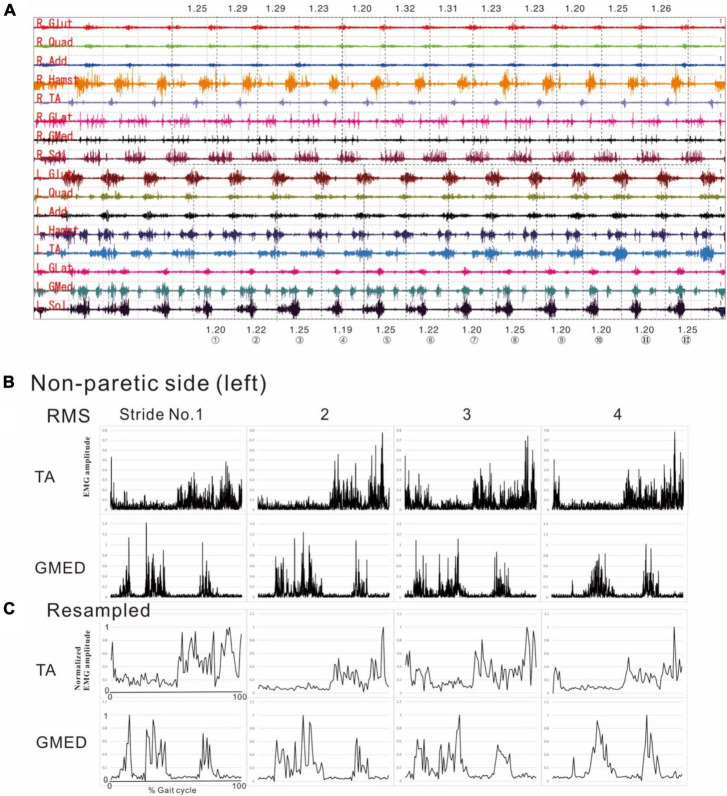
Methods of electromyographic (EMG) analysis. **(A)** EMG recordings while the patient was walking (3 km/h) on a treadmill just before discharge from hospital. On a video displayed on Teraview^®^, one stride was demarcated as the segment from one heel strike (dotted line) to the next heel strike (dotted line). Strides Nos. 1 to 12 are numbered, and the stride time is shown in seconds. R; right side (paretic side), L; left side (non-paretic side), Glut; gluteus maximus, Quad; quadriceps femoris, Add; adductor femoris, Hamst; hamstrings, TA; tibialis anterior, GLat; lateral gastrocnemius, GMed; medial gastrocnemius, Sol; soleus. **(B)** Results of root-mean-square (RMS) calculated from the raw EMG data for the tibialis anterior (TA) and medial gastrocnemius (GMed) from the first to fourth stride on the non-paretic (left) side. **(C)** Results of resampling varied amounts of data with stride times ranging from 1.19 to 1.25 s/strike uniformly into 100 data. The RMS data in the middle panels **(B)** show the data of time (seconds) taken by each stride sampled at 2,000 Hz, while the data in the lower panels **(C)** show the data resampled to 100 (*X*-axis of first stride in C: 0 to 100). Amplitude is normalized by setting the maximum value for each muscle to 100. Although fine changes in the waveform are seen in the RMS data, these fine fluctuations have disappeared after resampling, after applying a high-cut (low-pass) filter.

The filters used to process surface EMG waveforms and the number of strides (number of samples) used in each analysis affect the results of muscle synergy analysis (Chujo et al., 2022). In previous reports, raw EMG waveforms were processed with a high-pass (low-cut) filter at 40 Hz; then the full-wave rectified waveforms were processed with a low-pass (high-cut) filter at 4 Hz (Ting and Macpherson, 2005; Allen et al., 2013; Kouzaki, 2019; Ishibashi et al., 2021; Chujo et al., 2022). Many of these reports converted the activities of many muscles involved in gait into numerical quantities for analysis (Table 2). In the present study, we calculated the root-mean-square (RMS) of the raw EMG data to obtain the rectified waveform. For the raw EMG waveform data obtained at a sampling frequency of 2,000 Hz, we converted the sampling frequency such that 1 stride contained uniformly 100 data (resampling^1^). By resampling, we averaged out the data with highly variable stride times (time taken for 1 stride). Using this method, we set the condition to be equivalent to a 40-Hz low-pass (high-cut) filter. Setting the low-pass filter at a frequency higher than that used in previous reports probably accounted for the fine fluctuations observed in the activity waveforms of muscle synergies. Furthermore, we normalized the amplitude of muscle activity during one stride by setting the maximum activity of each muscle to 100 (Figure 1). Matlab ver. 2022b (MathWorks, Natick, MA, USA) was used to perform RMS, resampling, and non-negative matrix factorization (NNMF^2^)*. Default parameters were used.

**TABLE 2:** Number of strides analyzed and filter used in selected reports.

References	Subjects	No. of strides	Filter (LPF and HPF)
Kautz et al., 2011	Stroke patients and healthy individuals	Average 8.5 steps	HPF at 40 Hz for EMG, followed by LPF at 4 Hz for smoothening
Allen et al., 2013	Stroke patients and healthy individuals	Not mentioned	High-pass (low-cut) filtered at 40 Hz followed by low-pass (high-cut) filtering after rectification
Oliveira et al., 2014	Healthy individuals	20 concatenated steps	Band-pass filtered at 20–500 Hz, full-wave rectified, then high-cut (low-pass) filtered at 10 Hz
Barroso et al., 2014	Healthy individuals	10 consecutive gait cycles	High-pass (low-cut) filtered at 20 Hz, fully rectified, then low-pass (high-cut) filtered at 5 Hz
Hashiguchi et al., 2016	Stroke patients	10 gait cycles	Band-pass filtered at 20–250 Hz, rectified, then low-pass filtered at 10 Hz
Tan et al., 2020	Stroke patients	Maximal number of strides collected	Band-pass filtered at 30–400 Hz, rectified, then LPF at 10 Hz
Ishibashi et al., 2021	Stroke patients	6 gait cycles	Band-pass filter at 20–450 Hz. High-pass (low-cut) filtered at 40 Hz, rectified, then low-pass (high-cut) filtered at 4 Hz
Chujo et al., 2022	Stroke patients	20 gait cycles	Band-pass filtered at 40 Hz, low-pass (high-cut) filtered at 4 Hz
Mizuta et al., 2022	Stroke patients	20 gait cycles	Band-pass filter at 20–500 Hz and cutoff filter at 10 Hz (LPF?)

**FIGURE 2:**
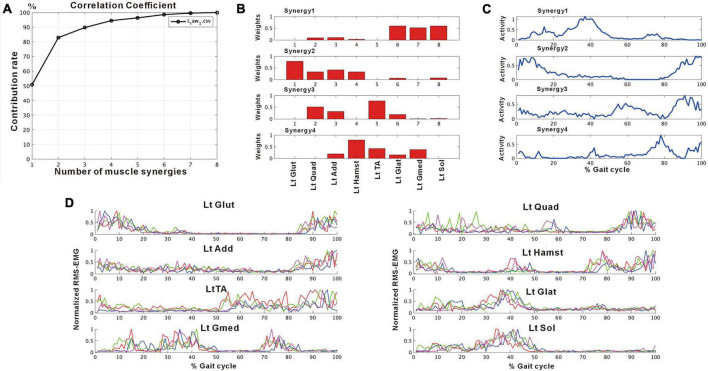
Results of non-negative matrix factorization (NNMF) analysis of the non-paretic side, analysis using four strides. **(A)** Correlation coefficient, **(B)** weights of each muscle synergy, **(C)** activities of each muscle synergy, **(D)** root-mean-squared EMG. Numbers in the weight plots **(B)** represent different muscles, the names of which are shown in the table in the lowest row. In this study, the four muscle synergies observed on the non-paretic side are considered to be the basic model. Syn-1 shows increased activity during late stance phase and the triceps surae is strongly involved. Syn-2 shows increased activity in the first half of stance phase and at the next heel strike, and lower limb proximal muscles are strongly involved. Syn-3 shows increased activity in early swing phase and the tibialis anterior is prominently involved. Syn-4 shows increased activity in late swing phase and the hamstrings are mainly involved. In the case of three muscle synergies, in addition to Syn-1 Syn-2, the third muscle synthesis is Syn-3 or Syn-3 + 4 (a pattern showing integration of Syn-3 and Syn-4). The order of Syn-1 and Syn-2, and that of Syn-3 and Syn-4 may be reversed.

Figure 2 shows the results of analysis of the patient’s non-paretic lower limb. As will be described later, analysis conducted using 4 strides per analysis yielded four muscle synergies in some analyses and three in others. The variance accounted for (VAF) between the raw data of the measured EMG and the product of two matrices obtained by approximate factorization using NNMF analysis is expressed as correlation coefficient in this study. The number of muscle synergies required for the gait task was defined as the minimum number of synergies with correlation coefficient above 90% (Figure 2A).

**FIGURE 3:**
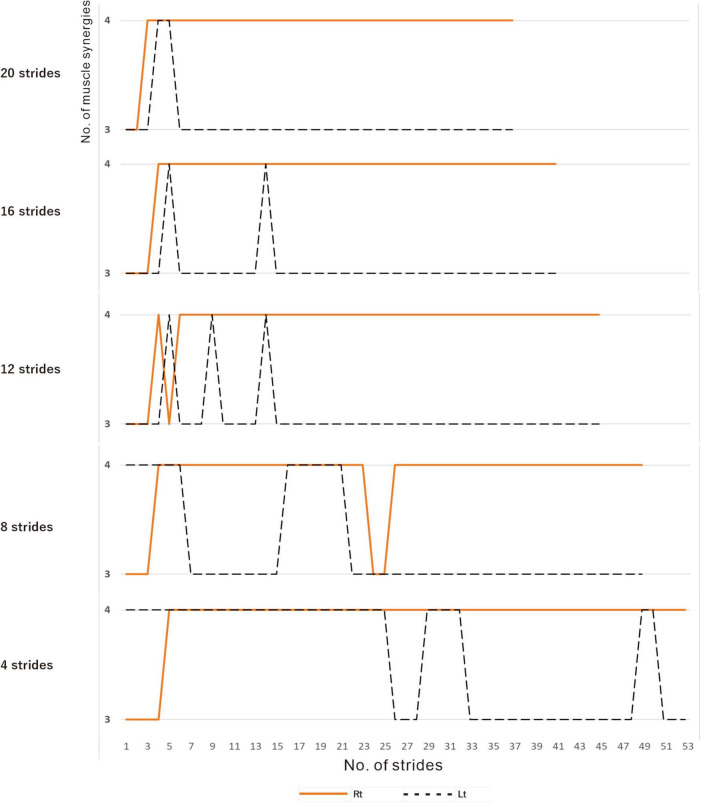
Relationship between the number of strides analyzed and the number of muscle synergies extracted. Results of analyses of treadmill walking just before discharge. A recording of 60 strides during walking at 3 km/h was available. For the analyses using four strides, the initial four strides from 1 to 4 were first analyzed, followed by the next four strides from 2 to 5, and so on. For the analyses using 20 strides, 20 strides were analyzed consecutively, first from 1 to 20 followed by 2 to 21 steps, and so on. On the paretic side (right, orange solid line), four muscle synergies were observed, except at the beginning of walking, regardless of the number of strides analyzed (4, 8, 12, 16 or 20 strides). On the non-paretic side (left, black dotted line), three muscle synergies were observed in the analysis using 20 strides, except at the beginning of walking (top row). When the number of strides analyzed was gradually reduced to 16, 12 and 8, four muscle synergies were observed at increasing frequencies. In the analysis using four strides each, four muscle synergies were observed in the first half of the 60-stride walk, and then shifted to three muscle synergies at around the middle of the walk (bottom row).

In muscle synergy analysis, apart from the filter setting for processing the raw EMG data, there is also a debate on how many strides should be used in one analysis set (Table 2). Chujo et al. (2022) found that 20 strides (gait cycles) were required to obtain a stable number of muscle synergies during walking in chronic post-stroke patients. In our case, since the number of muscle synergies was three or four depending on the number of strides analyzed (number of samples), we investigated the optimal number of strides for use in muscle synergy analysis (Figure 3). EMG recordings were done four times during hospitalization (Table 1), and the number of analyzable strides in each recording was limited, with a maximum of 60 strides in the last recording. As in the calculation of the moving average, analysis was performed by shifting the starting stride number forward by one when sampling the strides for analysis. On the paretic side (right lower limb, solid orange line in Figure 3), four muscle synergies were detected regardless of the number of strides analyzed (4 to 20 strides), except at the start of walking. On the non-paretic side (left lower limb, black dashed line in Figure 3), three muscle synergies were detected when 20 strides were analyzed, and the frequency of extracting four muscle synergies increased progressively as the number of strides was reduced to 16, 12, 8 and 4. From the observation that an increase in muscle synergy reflects improvement of gait ability (Routson et al., 2013) and for the purpose of examining the possibility of compensatory responses occurring on the non-paretic side during recovery of the paretic limb, we analyzed the changes in number of muscle synergies using 4 or 8 strides in one analysis set, which have been reported to allow sensitive observation of the changes (Oliveira et al., 2014).

### 3.4 Methodology of gait analysis

**FIGURE 4:**
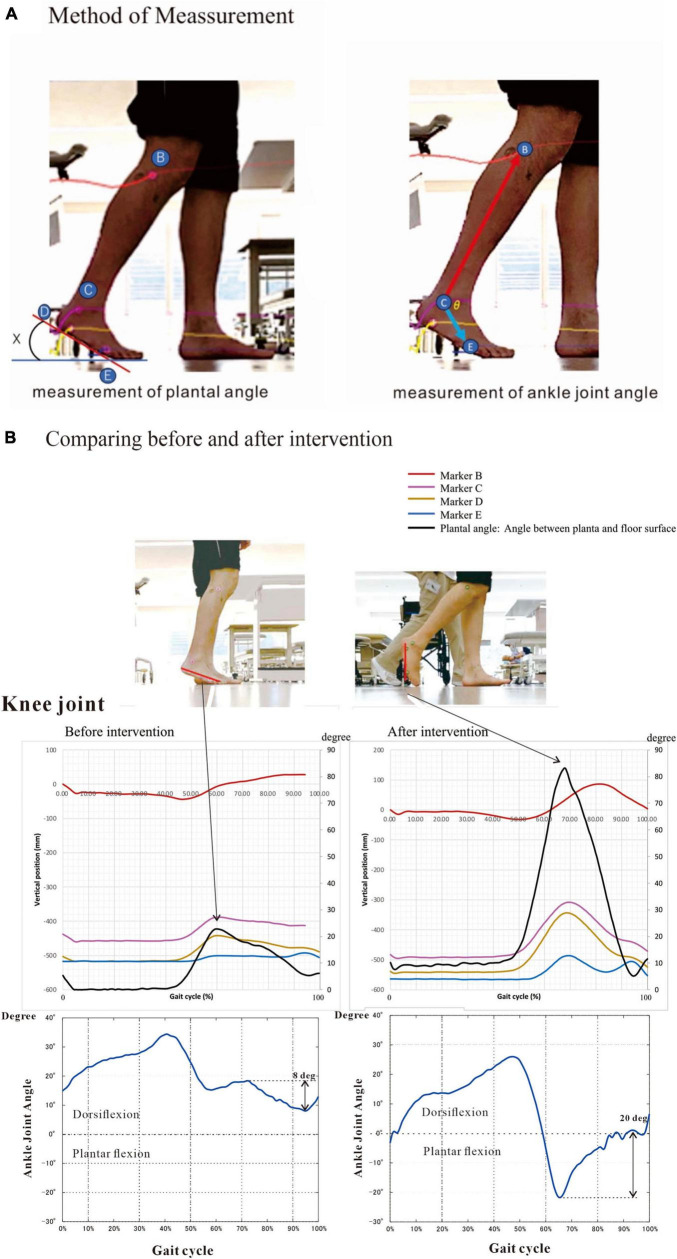
Method and results of kinematics analysis. **(A)** Angle between planta and floor surface and ankle joint angle were calculated by the changes in marker position during flat floor walking. Markers were placed on the head of fibula (B), the lower end (D) of the lateral malleolus (C) and the head of the fifth metatarsal bone (E). The equations are shown below. **(B)** We measured kinematics three times during hospitalization. Here we present the initial and the last measurements. At the last examination (at discharge), plantar angle was increased four times compared with that on admission. This angular change was probably due mainly to the increased knee joint flexion. The ankle joint dorsiflexed about 20 degrees at the terminal swing phase at discharge, compared to about 8 degrees of plantar flexion before intervention. To precisely compare the changes in kinematics, we measured under the same condition of barefoot walking. Angle (X) is calculated by the following equation: X (degree) = arcsin [difference (mm) in height between D and E/distance (mm) between D and E] The positions of marker B, C, D and E are described as B(x_*n*_, y_*n*_), C(x_*n*_, y_*n*_), D(x_*n*_, y_*n*_) and E(x_*n*_, y_*n*_), respectively. The length of the foot between D and E was Dist (mm). Then, X_*n*_ = arcsin[(D(y_*n*_)-E(y_*n*_))/Dist] The ankle joint angle (θ) is calculated by the following equations: $\vec{\text{CB}}$ = vector[B(x_*n*_)-C(x_*n*_), B(y_*n*_)-C(y_*n*_)] = $\vec{\text{a}}$ $\vec{\text{CE}}$ = vector[E(x_*n*_)-C(x_*n*_), E(y_*n*_)-C(y_*n*_)] = $\vec{\text{b}}$ $\vec{\text{a}}$ • $\vec{\text{b}}$=|$\vec{\text{a}}$| |$\vec{\text{b}}$| cosθ: Inner product of vector a and b θ = arccos[$\vec{\text{a}}$ • $\vec{\text{b}}$/|$\vec{\text{a}}$| |$\vec{\text{b}}$|].

Upon analyzing muscle synergies focusing on muscle activity, we analyzed the effects of muscle synergies on joint movements as output using the following method. Markers were placed on the base of the lateral malleolus (presumed to be the contact point of the calcaneus with the floor) and the head of the fifth metatarsal. Then, the walking movements were videorecorded, and the changes in position of the markers were recorded. The changes in muscle activities and their effects on joint angles were analyzed. For analysis, the angle of the foot to the floor was obtained by joining the two points of the markers as the hypotenuse and calculating the arcsin (Figure 4A).

Angle (X) between planta pedis and floor surface is calculated by the following equation:

X (degree) = arcsin [difference (mm) in height between D and E/distance (mm) between D and E]

The positions of marker B, C, D and E are described as B(x_*n*_, y_*n*_), C(x_*n*_, y_*n*_), D(x_*n*_, y_*n*_) and E(x_*n*_, y_*n*_), respectively. The length of the foot between D and E was Dist (mm).

Then, X_*n*_ = arcsin[(D(y_*n*_)-E(y_*n*_))/Dist]

The ankle joint angle (θ) is calculated by the following equations:

$\vec{\text{CB}}$ = vector[B(x_*n*_)-C(x_*n*_), B(y_*n*_)-C(y_*n*_)] = $\vec{\text{a}}$

$\vec{\text{CE}}$ = vector[E(x_*n*_)-C(x_*n*_), E(y_*n*_)-C(y_*n*_)] = $\vec{\text{b}}$

$\vec{\text{a}}$ • $\vec{\text{b}}$=|$\vec{\text{a}}$| |$\vec{\text{b}}$| cosθ: Inner product of vector a and b

θ = arccos[$\vec{\text{a}}$ • $\vec{\text{b}}$/|$\vec{\text{a}}$| |$\vec{\text{b}}$|]

The data were recorded with an iPhone and analyzed using the motion analysis software Kinovea ver. 0.8.27.^3^ The kinematic analyses were done three times (Table 1).

## 4 Results

After approximately 75 days of in-hospital rehabilitation training, the patient’s walking stability and endurance improved, and he was discharged from the hospital and subsequently returned to his former job. However, he exhibited automatic-voluntary dissociation and remained unable to voluntarily contract individual lower limb muscles, especially the tibialis anterior and triceps surae (Table 1).

### 4.1 Similarity of muscle synergies

We confirmed that the typical composition of the four patterns of muscle synergies obtained by our method was similar to previous reports (Clark et al., 2010; Chujo et al., 2022). The naming and characteristics of the muscle synergies are described below:

Synergy-1 (Syn-1): The activity profile showed that the activity of this synergy increased during late stance phase, and muscle weighting plot showed that the triceps surae was primarily involved.

Synergy-2 (Syn -2): In this synergy, activity increased in initial stance phase and at the next heel strike. Weighting showed that the lower limb proximal muscles (gluteus maximus, quadriceps, adductors, and hamstrings) were mainly involved.

Synergy-3 (Syn-3): In this synergy, activity increased in initial and terminal swing phase, and the tibialis anterior was predominantly involved.

Synergy-4 (Syn-4): In this synergy, activity increased at terminal swing, and the hamstrings were mainly involved.

The characteristics of Syn-1 and Syn-2 did not differ irrespective of whether three or four muscle synergies were detected, but the order of Syn-1 and Syn-2 or that of Syn-3 and Syn-4 was reversed in some analyses. In some analyses with three muscle synergies, Syn-3 showed a pattern resembling a combination of Syn-3 and Syn-4 seen in analyses with four muscle synergies; this pattern of muscle synergy was named Synergy-3 + 4 (Syn-3 + 4).

### 4.2 Changes in muscle synergies during process of recovery

#### 4.2.1 Early stage of hospitalization (10 days after admission, 33 days after onset)

**FIGURE 5:**
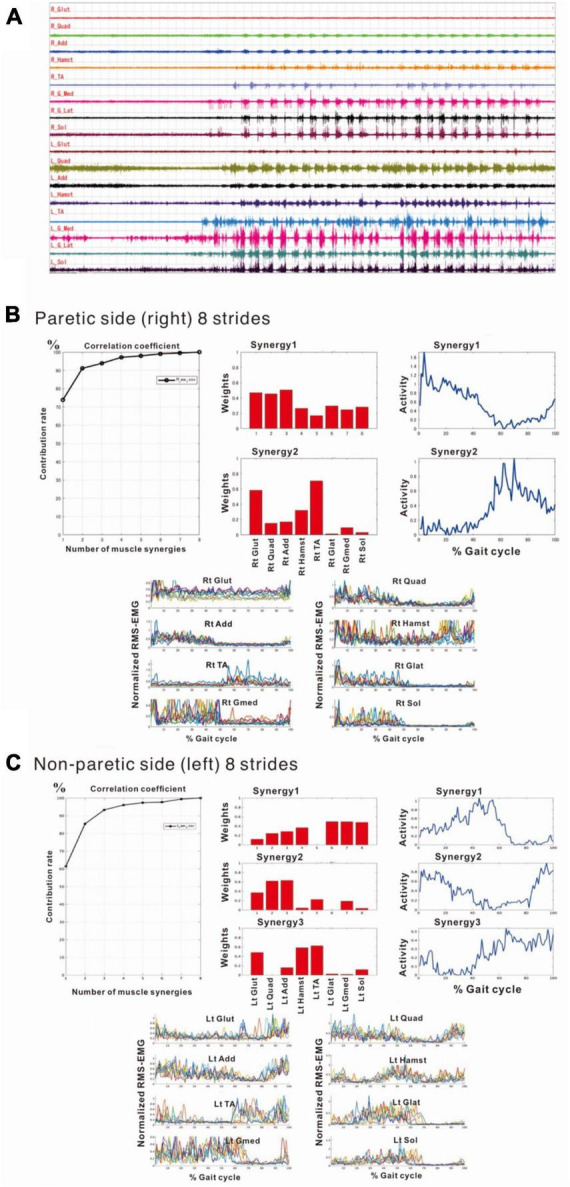
Surface electromyographic (EMG) recordings during walking on the flat floor at 10 days after admission. **(A)** EMG recordings of walking on the floor of the rehabilitation room (Nagaoka et al., 2022). In the right lower limb, despite the difficulties to voluntarily contract individual muscles, muscle activities consistent with the rhythm of walking are recorded on the paretic (right) side, although the interference waveforms are inferior to those recorded on the non-paretic (left) side. The NNMF analysis extracts two muscle synergies on the paretic side and three on the non-paretic side, and it is possible to explain the original waveform with a probability over 90%. **(B)** Results of NNMF analysis of the paretic side. The plots at the far right show the activities in one gait cycle. Two muscle synergies; Syn-1 activated in stance phase (first 50% of the gait cycle) and Syn-2 activated in swing phase, are extracted. For Syn-1, the weights of muscles involved in muscle synergies are high for gluteus maximus (labeled 1 on *X*-axis), quadriceps femoris (2) and adductor (3); but hamstrings (4), tibialis anterior (5), lateral gastrocnemius (6), medial gastrocnemius (7) and soleus (8) are also involved. For Syn-2, gluteus maximus (1) and tibialis anterior (5) are prominently involved. **(C)** Results of NNMF analysis of the non-paretic side. Syn-2 with high activity in late stance, Syn-1 with increased activity in early stance and at the next heel strike, and Syn-3 with high activity in swing phase are extracted. As for the weighting of muscles involved in synergy, high involvement is observed for triceps surae in Syn-1, femoris muscle group in Syn-2, and tibialis anterior and hamstrings in Syn-3.

Compared with the surface EMG of the non-paretic (left) side, those on the paretic (right) side showed fewer interference waveforms for each muscle (Figure 5A; Nagaoka et al., 2022). In this stage, the NNMF analysis of 8 strides detected two muscle synergies on the paretic side (three muscle synergies in the analysis of 4 strides), and three on the non-paretic side. It was possible to explain the original EMG waveform with a probability of 90% or above (VAF plots in Figures 5B, C). The NNMF analysis of the paretic side extracted one muscle synergy activated during stance phase in the first 50% of the gait cycle and the other muscle synergy activated during the swing phase in the latter 50% (activity plot in Figure 5B). Weighting data of the muscles involved in Syn-1 (weighting plot in Figure 5B; the numbers on the *X*-axis denote different muscles) showed high weights for the gluteus maximus (1), quadriceps femoris (2), and adductor (3); although the proximal muscles such as hamstrings (4), tibialis anterior (5), lateral gastrocnemius (6), medial gastrocnemius (7), and soleus (8) had relatively low weights, all muscles were involved. For Syn-2, the gluteus maximus (1) and tibialis anterior (5) were primarily involved.

The NNMF analysis of the non-paretic side extracted Syn-1 with high activity in late stance phase, Syn-2 with increased activity in early stance phase and at the next heel strike, and Syn-3 + 4 showing high activity in swing phase (Figure 5C). From the weighting data of the non-paretic side, the muscles prominently involved were the triceps surae in Syn-1, the femoris muscle group in Syn-2, and the tibialis anterior and hamstrings in Syn-3. These findings were consistent with the typical patterns reported previously.

#### 4.2.2 During training (24 and 54 days after admission, 47 and 77 days after onset)

In the second analysis, we noticed that the patient tended to have shorter strides due to the tendency of knee flexion in the latter half of the gait cycle. In this analysis, EMG of the right gluteus maximus was missing, and NNMF was done on seven muscles excluding gluteus maximus. EMG analysis always showed a three-synergy pattern (Figure 11, left without AFO, and Figure 9) when 4 or 8 strides were analyzed. Syn-1 was proximal dominant, and Syn-2 was distal muscle dominant, but these patterns were immature compared with the typical pattern in Figure 2. Syn-3 started to be activated in the late stance and early swing phase, and TA had the greatest weight. Based on the clinical observation and EMG findings, we started to use IVES (Muraoka et al., 2013) to increase muscle power, particularly in quadriceps femoris and tibialis anterior.

**FIGURE 6:**
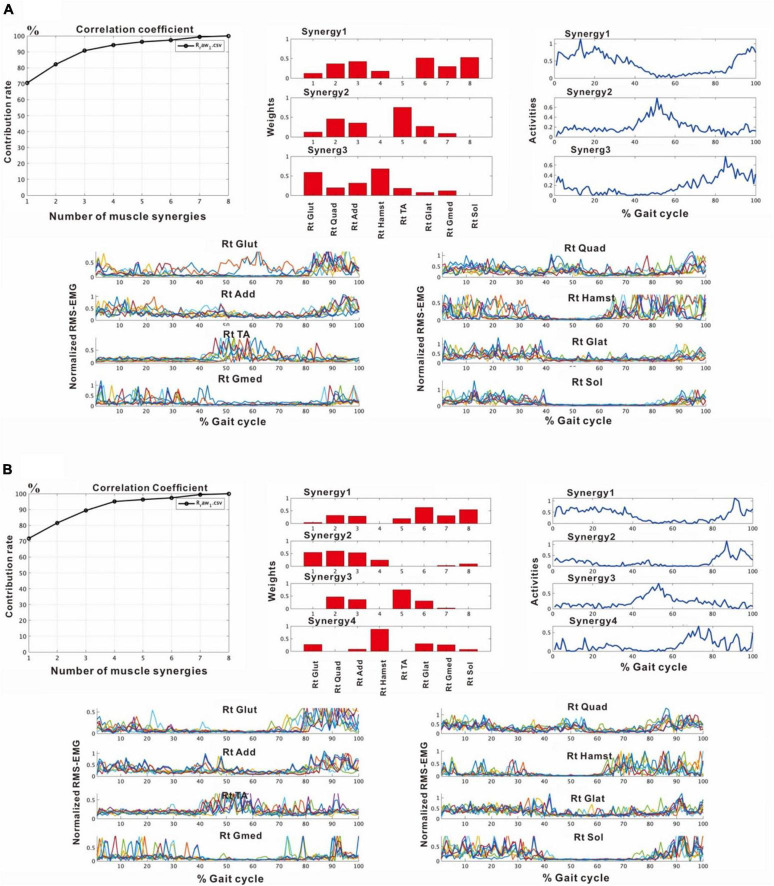
Results of analyses of the paretic (right) side during rehabilitation training (54 days after admission), using 8 strides per analysis. **(A)** Shows the analysis in which three muscle synergies are detected. From the recordings of activities shown on the far right, Syn-1 involving the lower limb extensor muscles in later half of stance phase, Syn-3 involving the tibialis anterior in early swing phase, and Syn-2 involving the lower limb proximal muscles in early stance phase and at the next heel strike are observed. **(B)** Shows the analysis in which four muscle synergies are detected. From the activity recordings, Syn-1 involving the lower limb extensor muscles in late stance phase, Syn-2 involving the femoris muscle group in early stance phase, Syn-3 involving the tibialis anterior in early swing phase, Syn-4 involving the hamstrings in late swing phase can be discriminated.

In the third analysis, the number of extracted muscle synergies changed depending on the number of strides (number of samples) used in one analysis set. In the analysis of the paretic side using 8 strides, two patterns, one with three muscle synergies and the other with four muscle synergies were obtained (Figure 6). When three muscle synergies were extracted, they consisted of Syn-2 involving the lower limb distal muscle group, Syn-3 involving the tibialis anterior, and Syn-1 involving the femoris muscles (Figure 6A). When four muscle synergies were detected, they comprised Syn-1 involving the proximal muscles, Syn-2 involving the triceps surae, Syn-3 involving the tibialis anterior, and Syn-4 involving the hamstrings (Figure 6B). However, these findings of the paretic side differed slightly from those on the non-paretic side (Figure 2) with respect to muscle synergies, activity, and muscle weighting. As a result, the flexion-dominant pattern in the second analysis was improved in the third analysis. The changes in synergy patterns are shown in Figures 6, 11.

#### 4.2.3 Before discharge (72 days after admission, 3 months after onset)

**FIGURE 7:**
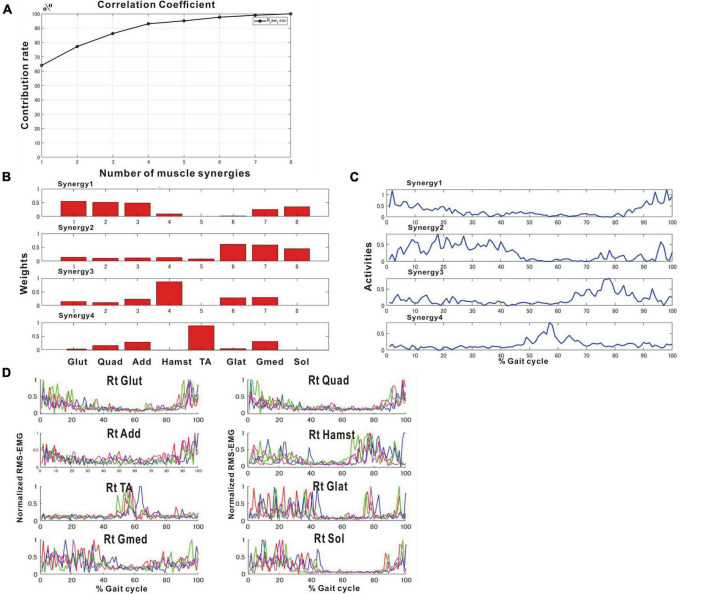
Results of analysis just before discharge (72 days after admission). **(A)** Correlation coefficient, **(B)** weights of each synergy, **(C)** activities of each synergy, **(D)** root-mean-squared EMG. **(B,C)** The four muscle synergies on the paretic side consist of Syn-2 with mainly the lower limb proximal muscles activated at initial contact and the next heel strike, Syn-1 with primarily the triceps surae activated at midstance, Syn-4 with the femoral flexors (hamstrings) activated at late swing phase, and Syn-3 with the tibialis anterior activated at early swing phase. In this analysis, the order of Syn-1 and Syn-2 and the order of Syn-3 and Syn-4 are reversed compared to the order shown in the plots of Figure 2 that represent the model pattern of this study.

After approximately 2 months of in-hospital training, the patient was able to walk outdoors independently, but he decided to use an AFO to improve endurance in preparation for returning to his former job that involves working mainly in a standing position. In this stage, the results of muscle synergy analysis conducted without wearing orthosis yielded mainly four muscle synergies on the paretic side (Figure 7) and three or four muscle synergies on the non-paretic side.

**FIGURE 8:**
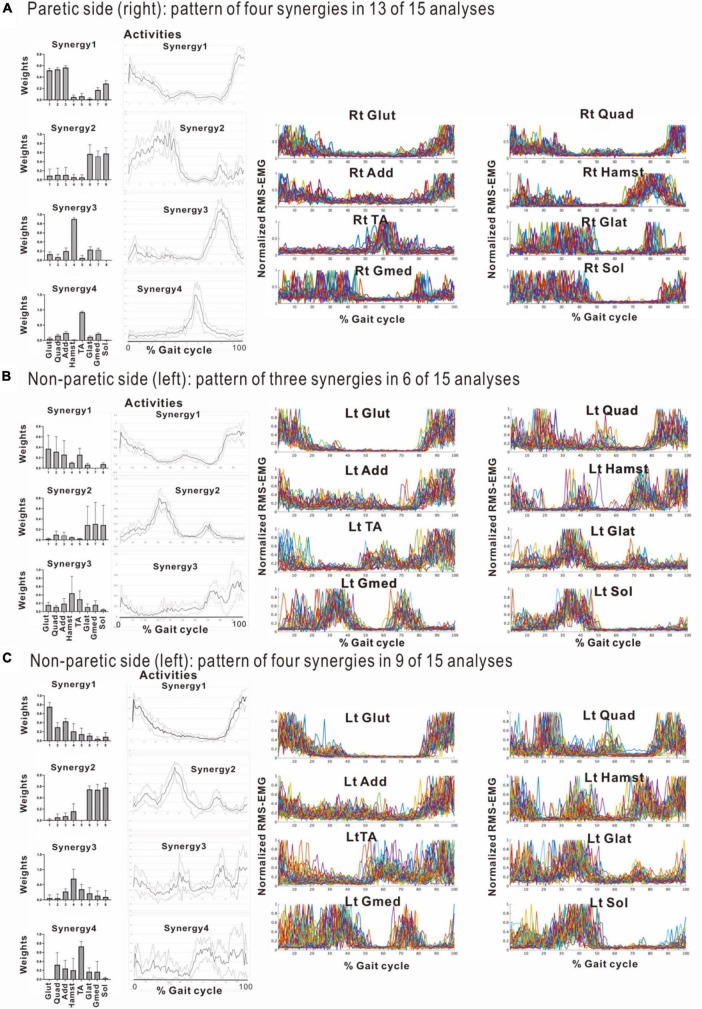
Comparison of stabilities of muscle synergies between paretic and non-paretic side recorded before discharge (72 days after admission). The weighting and activity data of the muscles constituting the four synergies on the paretic side **(A)** as well as four or three synergies on the non-paretic side **(B,C)** are expressed in mean and standard deviation. The standard deviations are small on the paretic side. On the non-paretic side, the standard deviations of Syn-1 and Syn-2 are small, but those of Syn-3, Syn-4, and Syn-3 + 4 tend to be large. The number of samples of the paretic side was *n* = 13, and the numbers of the non-paretic side were *n* = 9 for four-synergy analysis, and *n* = 6 for three-synergy analysis.

**FIGURE 9:**
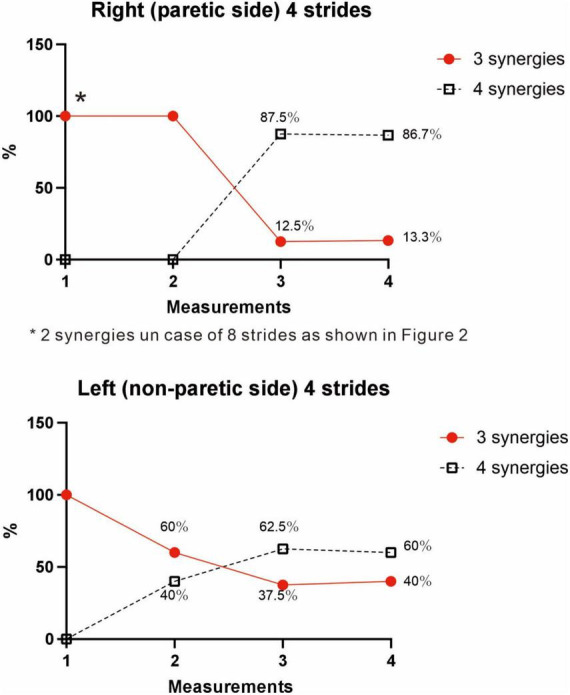
Changes in number of muscle synergies during the course of rehabilitation training: analyses using 4 strides. Measurements were performed four times during the hospital stay: (1) 10 days after admission (18 strides), (2) 24 days after admission (20 strides), (3) 54 days after admission (32 strides), and (4) 72 days after admission (before discharge) (60 strides). Unlike the method in Figure 3, calculation was performed for each group of 4 consecutive steps without overlapping. In the 4th measurement, 60 strides can be analyzed, and the results of 15 analysis sets are plotted. In Measurement No. 1, the paretic side shows two synergies using 8 strides, but 3 synergies using 4 strides. The frequency of detecting four synergies increases during the course of training, with 13 of 15 analyses extracting four synergies at the time of discharge. Likewise, the non-paretic side shows three synergies initially, and the frequency of detecting four synergies increases over time, with 9 of 15 analyses yielding four synergies before discharge.

The four muscle synergies on the paretic side consisted of Syn-2 with mainly the lower limb proximal muscles activated at initial contact and the next heel strike, Syn-1 with primarily the triceps surae activated at midstance, Syn-4 with the femoral flexors (hamstrings) activated at late swing phase, and Syn-3 with the tibialis anterior activated in early swing phase (Figures 7B, C). When a total of 60 strides were analyzed with a sample size of 4 strides in each analysis set, the results of 15 analysis sets were obtained. On the paretic side, four muscle synergies were observed in 13 of 15 analyses (86.7%), and three muscle synergies in 2 analyses (13.3%) (Figures 8, 9).

On the non-paretic side, the number of muscle synergies extracted differed depending on the number of strides (number of samples) analyzed. Three muscle synergies were extracted when using 12 or 8 strides in one analysis set, while three muscle synergies were extracted in some analyses and four in others when using 4 strides (Figure 9).

#### 4.2.4 Changes in muscle synergies in paretic lower limb in the course of recovery

At the start of training, three muscle synergies were observed in analyses using 4 strides (two muscle synergies extracted in analyses using 8 strides). As gait improved, the number of analyses showing three muscle synergies decreased, and the number of analyses showing four muscle synergies increased (Figure 9). The characteristics of the muscle synergies obtained in the course of recovery were not the same as the four stable patterns observed just before discharge.

### 4.3 Muscle synergies on the non-paretic side

At the start of training, three muscle synergies were detected on the non-paretic side (left), comprising Syn-1 involving the triceps surae activated in late stance phase, Syn-2 involving left lower limb proximal muscles activated in early stance phase and at the next heel strike, and Syn-3 + 4 involving the hamstrings and tibialis anterior (Figure 5C).

We examined the relationship between the number of samples used and the number of synergies extracted, utilizing the measurement conducted just before discharge. As shown in Figure 3, three muscle synergies were extracted in the analyses using 20, 16, and 12 strides, while the number of analyses extracting four muscle synergies increased when 8 or 4 strides were used. Therefore, the number of muscle synergies varied depending on the number of strides analyzed. In addition, when comparing the results of the four measurements conducted during the recovery process after admission, the number of 4-stride analyses extracting three muscle synergies decreased, whereas the number yielding four muscle synergies increased (Figure 9).

**FIGURE 10:**
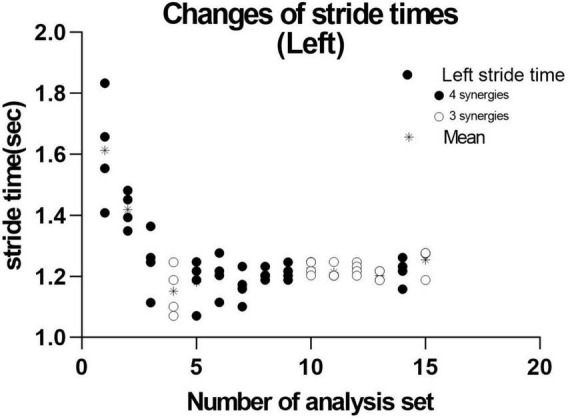
Distribution of stride time in non-paretic (left) lower limb. When analysis was conducted using 4 strides, the results of 15 analysis sets were obtained. The distribution and mean (*) stride time of the four strides used in each analysis extracting three (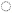) or four synergies (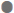) are plotted.

We next investigated the reason for extracting three or four muscle synergies on the non-paretic side using the measurement conducted just before discharge. Analyses were performed after excluding the data of the first 8 strides after the start of walking. First, gait symmetry was evaluated by comparing the stride times (the duration from heel strike of one foot to the next heel strike of the same foot) of the right foot and the left foot. There was no difference between the two feet (1.252 s for the right foot and 1.248 s for the left foot, *n* = 52 strides, not significant). There was also no difference in stride time between three and four muscle synergies obtained on the non-paretic side (three synergies: 1.215 s, *n* = 24; four synergies: 1.205 s, *n* = 28). We next examined whether the stride time of the paretic (right) limb influenced the number of muscle synergies extracted on the non-paretic side, but found no significant difference (stride time of right side corresponding to three synergies: 1.224 s, *n* = 24; stride time of right side corresponding to four synergies: 1.213 s, *n* = 28). For the non-paretic (left) limb, after excluding the first 8 strides, the stride time from 9 to 36 strides (first half; mainly four synergies were observed) had significantly larger variance than that from 37 to 60 strides (latter half, mainly three synergies were detected) (F test, *P*-value = 0.0005). For the paretic (right) limb, the variance between the first half and the second half was not significantly different. In the analysis of 60 strides, we examined the timing of appearance of muscle synergies during the course of walking. Four muscle synergies tended to appear in the first half of the walk and three muscle synergies to appear in the latter half on the non-paretic side (Figure 10, data on the paretic side are not shown).

### 4.4 Weighting and activation of muscles constituting the muscle synergies

When three synergies or four synergies were extracted, the activation and weighting profiles of the muscles involved in synergy were almost the same for both Syn-1 and Syn-2. This relationship was examined by comparing mean and standard deviation of the weights of the muscles involved in activation (Figure 8). On the non-paretic side, standard deviation of Syn-3 + 4 was larger than those for Syn-1 and Syn-2 when three muscle synergies were detected (standard deviations were larger in Syn-3 and Syn-4 than in Syn-1 and Syn-2 when four muscle synergies were detected) (Figures 8B, C). On the paretic side, however, when four muscle synergies were detected, the standard deviations of all the muscle synergies, especially Syn-3 and Syn-4, were smaller than those on the non-paretic side (Figure 8A). In summary, before discharge from hospital, four muscle synergies were extracted on the paretic side, and the weights of the muscles involved in activation of the muscle synergies were less variable compared with the non-paretic side.

### 4.5 Changes associated with use of ankle-foot orthosis

**FIGURE 11:**
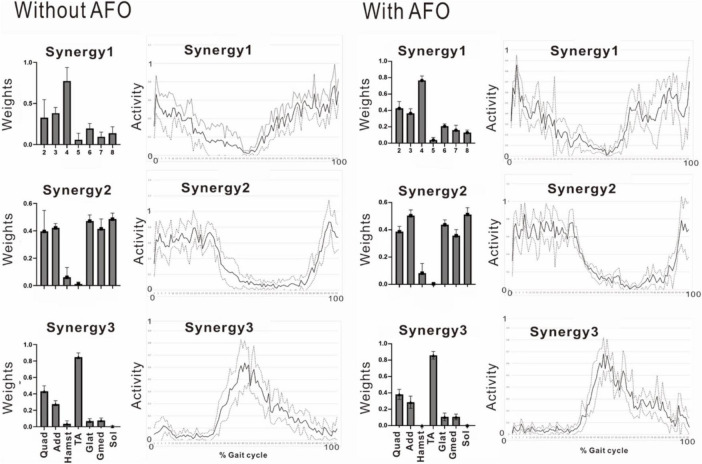
Effect of wearing ankle-foot orthosis: analysis of the paretic side 24 days after admission. EMG recording of right gluteus maximus was missing in this measurement. Then, NNMF was done on seven muscles excluding gluteus maximus. The patient performed treadmill walking barefoot but wearing an ankle-foot orthosis on the paretic side. In this stage, three muscle synergies are detected on the paretic side: Syn-1 with prominent activity of lower limb proximal muscles, Syn-2 with prominent activity of triceps surae, and Syn-3 with activity of tibialis anterior in early swing phase. No significant changes are observed with the use of an ankle-foot orthosis.

The patient used an AFO during the course of rehabilitation training. Although an AFO can be expected to assist ankle dorsiflexion controlled by the tibialis anterior during early swing phase, the analysis conducted 24 days after admission showed that wearing an AFO exerted no significant effect on the tibialis anterior muscle activity during the gait cycle (Figure 11).

### 4.6 Gait analysis

Markers were placed on the lower end of the lateral malleolus and the head of the fifth metatarsal bone, and walking movements were videotaped to record changes in position of the markers. At the beginning of rehabilitation training, the hypotenuse had a maximum angle of 25.6 degrees to the vertical downward direction (58.4% of the gait cycle). Just before discharge, however, the maximum angle became 83.1 degrees (63.9% of the gait cycle), suggesting strengthening of knee joint flexion in the swing phase on the paretic side (Figure 4B). In summary, changes in the EMGs infer that rehabilitation training increased the flexor activity of the paretic knee joint in early swing phase (Figures 6, 11) and the gluteus maximus muscle activity of the non-paretic limb, consequently changing the weights of the muscles that constitute the muscle synergies, while gait analysis confirmed that the training increased knee joint flexion during the swing phase in the paretic lower limb.

As Syn-3 was developed as training progressed, we expected the ankle joint to dorsiflex during the swing phase. At discharge, the ankle joint dorsiflexed about 20 degrees at the terminal swing phase (Figure 4B, bottom right), compared to plantar flexion of about 8 degrees before intervention (Figure 4B, bottom left). By muscle synergy analysis, four synergies, Syn-1 to 4, were extracted at discharge. Syn-3 with prominent weight at TA would be functioning in the late swing phase.

## 5 Discussion

In our patient with right lower limb monoplegia following left frontal lobe infarction caused by a dissecting aneurysm of the anterior cerebral artery, we recorded surface EMG of muscles in both lower limbs while walking during the course of recovery (stride length: from 986.3 to 1,456.6 mm, cadence: from 92.4 to 105.6 step/min), and analyzed the dynamics of muscle synergies associated with gait by NNMF. Regarding the muscle synergies involved in gait, many studies observed four muscle synergies in the unaffected limb or in healthy individuals. As shown in the results of the present case, the number of muscle synergies changed from the beginning of rehabilitation to before discharge. On the paretic side, two or three muscle synergies were observed at the beginning of training, increasing to four as gait was stabilized. On the non-paretic side, three muscle synergies were observed initially, but reducing the number of strides used in the analysis set yielded four synergies in some analyses and three in others. Our finding that the number of muscle synergies increased as gait parameters improved by rehabilitation training was consistent with previous reports (Clark et al., 2010, Hashiguchi et al., 2016). On the other hand, when the non-paretic side is considered healthy, the smaller number of muscle synergies extracted on the non-paretic side contradicts with previous reports. Changes were observed not only in the number of synergies, but also in the weighting and activation of the muscles that make up the synergies both on the paretic and non-paretic sides, and also during the course of training.

### 5.1 Methods of analysis

Lee and Seung (1999) introduced NNMF, principal component analysis, and vector quantization as analytical methods for perceiving the whole by learning the parts of objects. Many studies have reported an association between changes in number of muscle synergies with deterioration or improvement of motor function. In our analysis, however, the number of muscle synergies changed depending on the number of strides analyzed. Although previous reports have indicated that the number of muscle synergies detected depends on the filter used and the number of strides analyzed, few reports except Chujo et al. (2022) have examined the appropriate number of strides to be analyzed (Table 1). In general, a larger number of strides should be analyzed when the purpose is to establish a stable trend (Chujo et al., 2022), while the number of strides should be reduced when the aim is to detect the presence or absence of changes (Oliveira et al., 2014; Figure 3). However, although using 4 strides in the present study established the presence of changes in the EMG waveform of each stride, whether it is appropriate to analyze less than 4 strides should be judged by the steadiness of the number of muscle synergies obtained from the results of NNMF analysis.

We used an analytical method different from previous reports, aiming to simplify the procedure and to allow on-site analysis. As shown in Figure 2, when four muscle synergies were detected on the non-paretic side and the paretic side, we observed Syn-1 in early stance phase and at the next heel strike with strong involvement of the lower limb proximal muscles, Syn-2 in late stance phase with involvement of the triceps surae, Syn-3 in early swing phase with involvement of the tibialis anterior, and Syn-4 in late swing phase with involvement of the hamstrings. Since these four patterns of muscle synergies are consistent with those described in previous studies, we consider that our analytical method is appropriate and valid. The presence of fine fluctuations in the activity waveforms is probably because the low-pass filter that was a result of resampling was set at 40 Hz, which differed from the conventional methods using low-pass filter set at 4 to 10 Hz (The results obtained in this study possibly depends on specific features of the methods used.).

For NNMF analysis in this study, we averaged the data of four or eight strides and fed the averaged data into NNMF. We examined whether these results using averaged data differ from those using concatenated dataset, using the data of the fourth analysis (before discharge). We performed NNMF using concatenated datasets of all the 60 strides and of four strides each for the 60 strides (Supplementary Data). In most analyses, for both the paretic and non-paretic sides, five synergies were extracted: synergy-1 to synergy-4 as obtained using averaged data, with a fifth synergy that was often fragment of synergy-1 or -2. Even with the large concatenated dataset of 60 strides, five synergies were extracted. Thus, the results of concatenated data analysis are similar to those obtained from averaged data analysis. From the practical point of view, it seems to be difficult to know precisely the roles of specific synergies in activities during a single gait cycle when using concatenated dataset. We therefore used averaged data in NNMF analysis in this study.

### 5.2 Changes in number of synergies during the recovery process

#### 5.2.1 Changes on the paretic side

Decreased number of synergies was related to impairment and was reported to be caused by merging of unaffected synergies (Cheung et al., 2012). Among the various reports on changes in the number of muscle synergies in paretic limbs, two showed increases in number of muscle synergies as recovery progressed in post-stroke hemiplegic patients (Clark et al., 2010; Hashiguchi et al., 2016). We also observed an increase from two (analysis using 8 strides) or three muscle synergies (analysis using 4 strides) at the start of rehabilitation training to four muscle synergies before discharge (Figure 9; analyses using four strides without overlap). Recordings of the paretic limb just before discharge showed that four muscle synergies were maintained in the majority (87%) of the gait observations. The functional recovery of our patient was supported by synergy analysis. To classify the extracted synergies by NNMF into the typical synergy types, integrating the patterns of weights and activities is necessary, because each synergy has specific roles in the gait cycle.

Stroke patients are known to have greater step length in individual steps and longer stride time in individual gait cycles compared with healthy individuals (Balasubramanian et al., 2009). However, the variability, expressed in standard deviation, for the weights and activities of muscles that constitute the muscle synergies in the paretic limb was smaller than that in the non-paretic limb (Figures 8A vs. C). There was also no difference in stride time between the paretic and the non-paretic sides.

Not only the numbers of synergy, but patterns of muscle weights and activities also changed during the patient’s hospitalization (Figures 5B, 6A, 7, 11-left). Although the natural recovery process after infarction cannot be ruled out, the effects of interventions through rehabilitation (Table 1 in this case) should be discussed. The influences of rehabilitation on muscle synergies have been reported in sit-to-stand motion analysis in stroke patients (Kogami, 2018; Yang et al., 2019; Wang et al., 2022). At the second examination, we noticed that our patient tended to have shorter strides because of the tendency of knee flexion in the latter half of the gait cycle. We used IVES (integrated volitional control electrical stimulation) (Muraoka et al., 2013) to increase the power of some muscles such as quadriceps femoris and tibialis anterior. In the third examination, the TA-dominant pattern (Figure 11-left) was changed to two synergies; TA-dominant in one and Hamstrings-dominant in the other (Figure 6A). This observation should be confirmed by further synergy and kinematic analyses as suggested by Wang et al. (2022).

#### 5.2.2 Changes on the non-paretic side

In the analyses using 4 strides, the frequency of extracting four muscle synergies was 60% on the non-paretic side. Despite being the healthy limb (non-paretic limb), the frequency of four muscle synergies was less than that observed in the paretic limb (87%) (Figure 9). Just before discharge, analysis of 60 strides of 4 consecutive strides each (Figure 10) detected four muscle synergies in the first half of treadmill walking, but the number of muscle synergies shifted to three in the latter half of the walk (Figure 10). This finding was observed because fewer strides were analyzed in this study to allow sensitive observation of the recovery process. Whether three or four muscle synergies are extracted is related to whether Syn-3 and Syn-4 that involve the tibialis anterior and hamstrings, respectively, are separated. As shown in Figures 8B, C, in the non-paretic limb, the extraction of Syn-3, Syn-4, and Syn-3 + 4 is presumably related to their large standard deviations in the activity waveforms compared with Syn-1 and Syn-2. Considering that the number of muscle synergies reflects the interaction of the muscle activities involved in walking, it is possible that while the paretic limb (Figure 8A) walks in a constant pattern that can be described as stereotype, the non-paretic limb may be executing compensatory responses such as balance adjustment (regulated by the corticospinal tract and supplementary motor cortex of the unaffected side) (Oliveira et al., 2014).

The functional role of synergies on the paretic and non-paretic sides may be different. On the paretic side, the patient was able to walk faster with four synergies after rehabilitation training than with two synergies at admission, although the patterns were stereotypic. On the non-paretic side, the four synergies may flexibly modify activities, providing dynamic adaptation to walking, such as balance control. Increase in number of synergies was related to functional recovery in our patient. However, temporal characteristics in muscle synergy activities could also be an indicator of recovery (Yang et al., 2019). This aspect must be examined in future studies.

### 5.3 Results of gait analysis

In gait analysis, the maximum angle formed by the line connecting the lower end of the lateral malleolus and the head of the fifth metatarsal bone with the floor surface (plantar angle) was 25.6 degrees soon after the start of training, but the maximum angle became 83.1 degrees at around 60% of the gait cycle just before discharge (Figure 4B upper). The ankle joint dorsiflexed about 20 degrees in the terminal swing phase at discharge, compared to about 8 degrees of plantar flexion before intervention (Figure 4B, bottom). The pattern of ankle joint angle during the gait cycle became similar to that of normal subjects. By muscle synergy analysis, Syn-3 with the prominent weight at TA would be functioning in the late swing phase.

These findings suggest increase of the knee joint flexion angle during the swing phase in the paretic limb. Although we did not perform extensive kinematic observations including hip joints and pelvis, a one-to-one correspondence between muscle synergy and kinematic synergy in gait has been reported (Esmaeili et al., 2022). Therefore, we consider that the establishment of Syn-3 and Syn-4 on the paretic side may have given rise to the kinematic results. However, there is also a possibility that the increased activities of the gluteus maximus and quadriceps femoris muscle group in the non-paretic limb in response to early swing of the paretic limb may also augment the lifting action of the pelvis and assist the knee joint flexion of the paretic limb (Figures 5C, 8C, compare the weights and EMG of these muscles).

### 5.4 Neural mechanisms of gait

The cerebral cortex, basal ganglia, cerebellum, brainstem, and spinal cord are involved in gait (Takakusaki, 2017). The present case can be considered to have isolated corticospinal tract damage caused by the focal frontal lobe lesion. Bernstein (1967) proposed that in implementing complex movements in humans, the brain does not control a large number of muscles individually, but control groups of muscles (muscle synergies) to execute various repertoires of movements. Therefore, analyzing muscle synergies during the process of gait recovery in our patient will provide important clues for understanding the neural mechanisms involved in walking.

Assuming that muscle synergies composed of motor neurons controlling gait-associated muscles exist in the spinal cord, there are several questions of how loss of the corticospinal tract function may affect the muscle synergies: whether muscle synergies of the paretic side can regain the same level of activities as the unaffected side even though the corticospinal tract functional loss persists; whether damage to contralateral corticospinal tract affects muscle synergies on the non-paretic side; and whether muscle synergies on the paretic and non-paretic sides achieve the same performance when the paretic side is repaired. We anticipate that the present study will provide some insights to answer the above questions.

In our previous report of this case, we presumed that the focal cerebral infarction in the left paracentral lobule was a complete infarct, destroying the motor cortex corresponding to the lower limb. Near-infrared spectroscopy (NIRS) measurement during treadmill gait showed an increase in oxyhemoglobin concentration near the right central sulcus, but no increase was observed near the left central sulcus corresponding to the lesion (Nagaoka et al., 2022). In healthy individuals, the cerebral cortex motor area is involved in voluntary contraction of individual muscles. Based on the neurological symptoms of this case, the motor cortex function of voluntary muscle contraction was impaired. Yet, the patient was capable of walking. We explained these observations by hypothesizing that walking is controlled by structures other than the cerebral cortex motor area. On the other hand, the increased blood flow near the right central sulcus may reflect a potential physiological response associated with gait (controlled by the non-paretic left lower limb) and over-activity of the unaffected (non-paretic) lower limb to compensate for the impaired balance caused by right lower limb paresis.

In the muscle synergy analysis of the paretic side, initially two muscle synergies were activated, but the number increased to three and finally to four as rehabilitation training progressed (Figure 9). Given that the infarct was complete and the corticospinal tract was interrupted from the paracentral gyrus, this case demonstrates that muscle synergy can be activated even when corticospinal tract function is lost and that it is possible to increase the number of muscle synergies through rehabilitation training. On the other hand, comparing the four muscle synergies observed before discharge, the standard deviations of activities were larger on the non-paretic side than on the paretic side (Figure 8), and NNMF analyses using a sample size of 4 strides detected four muscle synergies at a probability of 13/15 on the paretic side, compared with the non-paretic side that had four muscle synergies at the start of walking, but the number decreased to three from the middle of the 60 strides (Figures 8, 9, 10). These data may reflect the changes in EMG pattern during walking.

On the paretic side, the variability of activity level (standard deviation) was small and the number of muscle synergies was constant with little changes. Together with the fact that tendon reflexes in the paretic limb were slightly augmented during this stage, these observations may suggest that the characteristics of the muscle synergies on the paretic side may reflect a form of stereotype. Reports have shown that disruption of upper spinal control is associated with a decrease in standard deviation of motor unit activity (Shimazu et al., 1962; Tokizane and Shimazu, 1964) and decreases in coherence at 10–25 Hz and short-term synchronicity in EMG firing (Nielsen et al., 2008). In our case, as a result of compensation for the balance disturbance produced by the paretic lower limb exhibiting stereotype regardless of situation, the variability of muscle activity increases and muscle synergies change on the unaffected (non-paretic) side, which probably accounts for the increased standard deviations in activity level.

A limitation of this study is that we did not record biomechanical evidence to confirm the balance impairment caused by stereotype movements such as center of gravity sway on the paretic side. Therefore, the discussions regarding the difference in number of muscle synergies between the paretic and non-paretic sides remain hypothetical. In addition, we cannot rule out the possibility that the characteristics of muscle synergies observed in this report reflect the use of a low-pass filter at a higher frequency of 40 Hz. Nevertheless, although many reports on gait of hemiplegic patients focus on the gait pattern, muscle activity pattern, and number of muscle synergies on the paretic side, it is necessary also to consider the effect on the non-paretic side, as we have done in this study. Furthermore, the optimal number of strides for analysis needs to be examined individually according to the purpose of the research and the magnitude of changes.

In this study, we explored the neuromechanical mechanisms underlying the gait recovery process of a monoplegic patient by conducting muscle synergy analysis combined with kinematics. We investigated the new methodological approach for muscle synergy analysis, elucidating various key factors for obtaining optimal results. The findings have the potential to be used as indicators in gait training. The utility of our methods in investigating the process of regaining gait function should be validated with more patients in the future.

## Data availability statement

The original contributions presented in this study are included in this article/Supplementary material, further inquiries can be directed to the corresponding author.

## Ethics statement

The studies involving humans were approved by the Ethics Committee of Ishibashi General Hospital (2021-No. 6). The studies were conducted in accordance with the local legislation and institutional requirements. The participants provided their written informed consent to participate in this study. Written informed consent was obtained from the individual(s) for the publication of any potentially identifiable images or data included in this article.

## Author contributions

AE: Investigation, Data curation, Methodology, Software, Writing – review & editing. MH: Investigation, Methodology, Supervision, Writing – review & editing. YK: Investigation, Methodology, Supervision, Writing – review & editing. MN: Conceptualization, Investigation, Project administration, Writing – original draft, Writing – review & editing.

## Footnotes

1 In the RMS data (Figure 1B), fine fluctuations are observed in the waveforms, but these fine fluctuations disappear in the waveforms after resampling (Figure 1C), because high-cut (low-pass) filtering has been applied. If the original waveform has a sampling frequency of 2,000 Hz and the time taken for 1 step is 1.25 s, then the number of data is 2,500. When this waveform is resampled so that the number of data becomes 100, the resampling frequency X is calculated as: 2,000: 2,500 = X: 100, and X = 80 Hz. A low-pass (high-cut) filter of 1/2 × X, the Nyquist frequency, is applied by the Matlab program to prevent aliasing when normalizing high frequency waveforms at low frequency. That is, a 40-Hz low-pass filter is applied.

2 Here, 8 muscles are recorded at a sampling interval of 500 μs (sampling frequency 2,000 Hz). If each step takes *n* seconds, then a matrix with 2,000 × *n* rows (I) and 8 columns (J) (*X* = I × J) can be constructed. Let K be the base number from 1 to 8, the matrix is represented by the product of I × K and K × J, and K is calculated so as to minimize the difference from the measured matrix. The similarity between the measured matrix and the product of the matrices obtained by factorization was defined as the variance accounted for (VAF), and the minimum K value at which VAF exceeded 90% was defined as the number of muscle synergies required for the walking task.

3
https://www.kinovea.org/
