# Supplementary material for: Analysis of muscle synergy and gait kinematics during regain of gait function through rehabilitation in a monoplegic patient
Source: Front Hum Neurosci. 2024 Jan 9;17:1287675. doi: 10.3389/fnhum.2023.1287675 (PMC10803437; doi:10.3389/fnhum.2023.1287675)
Supplement: Supplementary file 1 [file Data_Sheet_1.docx]

**Supplementary data**

All the 60 analyzable strides of the fourth examination were analyzed as a concatenated dataset. Five synergies were extracted. The patterns of synergies from Syn-1 to Syn-4 in this analysis were the same as the patterns observed using averaged datasets. The stability of patterns was higher on the right (paretic) than on the left (non-paretic) side; this finding is the same as the averaged data. Syn-5 seems to be fragment of Syn-1 or an unidentifiable pattern.

The extracted numbers of synergies were always five even when the 60 strides were analyzed by dividing into segment of 4, 8, 12, and 16 strikes. We showed that there was variability in muscle weights and activities on the non-paretic side by reducing of the number of strides per analysis. With 4-stride analysis, there were five synergies and some irregularity of extracted synergy patterns on the left side; that is, the numbers of unidentifiable patterns and fragmented patterns (1Gl, 1Gm and 1S) were higher on the left side.

If there are changes in activity patterns of specific synergies during training, they would be useful findings for feedback of gait training. The time scale of activity should be 100%, so that we can know when a specific synergy is activated during the gait cycle.

The fragmented patterns were classified as follows:

1Gl: Resembles Syn-1, but weight of gastrocnemius lateralis was prominent

1Gm: Resembles Syn-1, but weight of gastrocnemius medialis was prominent

1S: Resembles Syn-1, but weight of soleus was prominent

2Gx: Resembles Syn-2, but weight of gluteus maximus was prominent

**
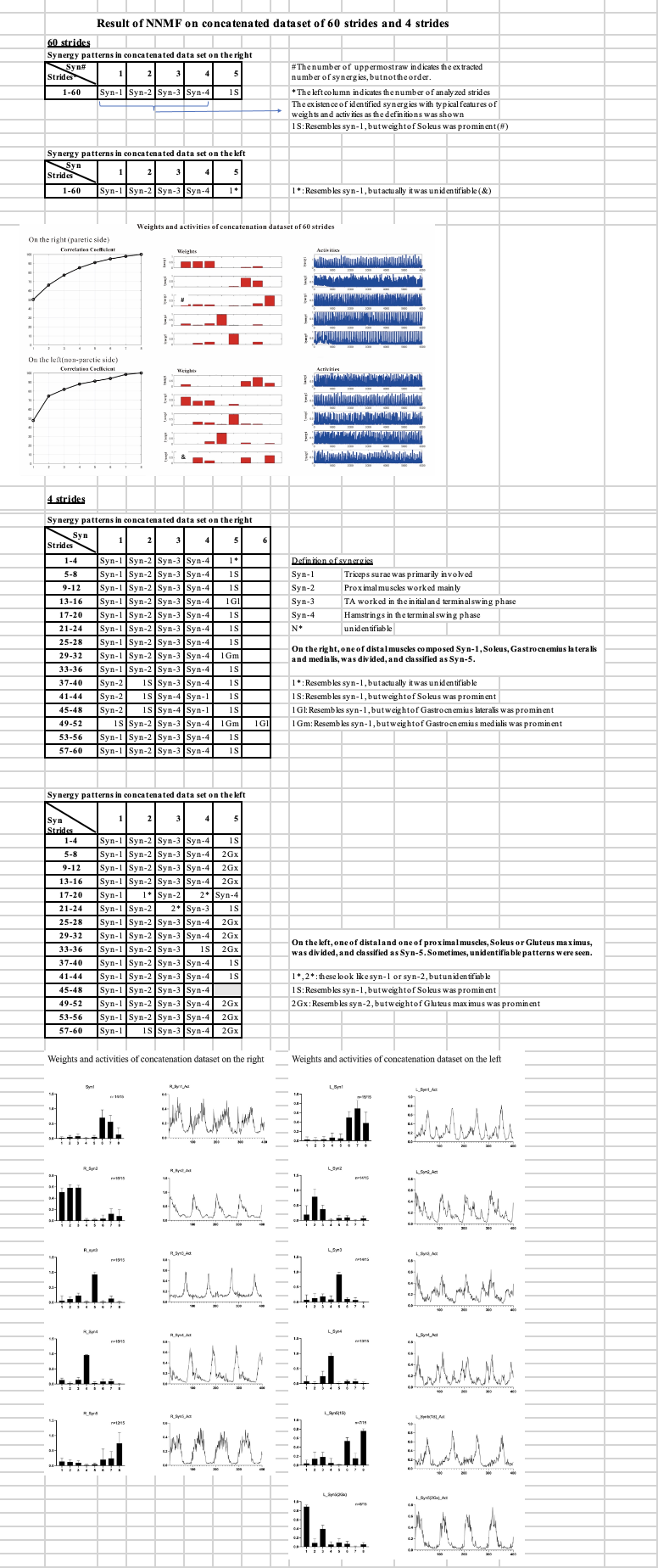
**
